# Forecasting the 2020 COVID-19 Epidemic: A Multivariate Quasi-Poisson Regression to Model the Evolution of New Cases in Chile

**DOI:** 10.3389/fpubh.2021.610479

**Published:** 2021-04-23

**Authors:** María Ignacia Vicuña, Cristián Vásquez, Bernardo F. Quiroga

**Affiliations:** Escuela de Administración, Pontificia Universidad Católica de Chile, Santiago, Chile

**Keywords:** coronavirus infections, forecasting, logistic models, Quasi-Poisson regression, non-linear dynamics, Chile

## Abstract

**Objectives:** To understand and forecast the evolution of COVID-19 (Coronavirus disease 2019) in Chile, and analyze alternative simulated scenarios to better predict alternative paths, in order to implement policy solutions to stop the spread and minimize damage.

**Methods:** We have specified a novel multi-parameter generalized logistic growth model, which does not only look at the trend of the data, but also includes explanatory covariates, using a quasi-Poisson regression specification to account for overdispersion of the count data. We fitted our model to data from the onset of the disease (February 28) until September 15. Estimating the parameters from our model, we predicted the growth of the epidemic for the evolution of the disease until the end of October 2020. We also evaluated via simulations different fictional scenarios for the outcome of alternative policies (those analyses are included in the Supplementary Material).

**Results and Conclusions:** The evolution of the disease has not followed an exponential growth, but rather, stabilized and moved downward after July 2020, starting to increase again after the implementation of the *Step-by-Step* policy. The lockdown policy implemented in the majority of the country has proven effective in stopping the spread, and the lockdown-relaxation policies, however gradual, appear to have caused an upward break in the trend.

## 1. Introduction

The pathogen SARS-Cov-2 has caused the infection called Coronavirus disease 2019 (COVID-19), spreading worldwide in just a few months. On January 30, 2020, the World Health Organization (WHO) declared the COVID-19 outbreak a *Public Health Emergency of International Concern*, considering the occurrence of cases in five WHO regions within 1 month (1).

Chile is an interesting case study to monitor the evolution of COVID-19: It is not a developed country, in spite of its membership in the Organization for Economic Co-operation and Development (OECD). Income inequality has been a persistent discussion topic for decades (2). Yet, its authorities took early measures to augment emergency room capacity and to restrict individual freedom of movement, in order to be able to face the pandemic.

On February 8, 2020, the Chilean government declared a national health emergency in the country, beginning Phase 1 of the epidemic “as no cases had been reported yet,” to deal with the imminent arrival of the virus. On March 3, 2020, the first case (an international traveler) was announced (3), meaning that the country had entered thus Phase 2 of the epidemic “all cases corresponding to people who had traveled abroad.” On March 6, 2020, the Chilean Ministry of Health (CMoH) issued a new legal order to increase its attributions to be able to mitigate the imminent local spread of the virus. Two weeks later (March 18, 2020), the Chilean government decreed a state of constitutional exception due to national catastrophe, after the WHO declared COVID-19 a pandemic on March 11 (1), enabling the government to restrict free movement and association. Some policies were implemented both at the national (curfews and prohibition of crowded events) and local (zonal weekly quarantines and regional sanitary blockages) levels, depending on the evolution of the disease (4).

By the end of June 2020, Chile had over two hundred and thousand confirmed cases of COVID-19. The majority of new cases concentrated in the Metropolitan Region (RM, where Santiago, the capital city, is located), where about a third of the country's population is concentrated. Chile has the highest testing rate in Latin America, with nearly one million tests carried out by June 23rd (4, 5). Overall case fatality was of 12,278 as of September 15th 2020.

Full lockdown in the RM was implemented on May 13th 2020. Progressive improvements in the daily number of both infected individuals and casualties, prompted the government to announce on July 16th a new policy called *Paso-a-Paso* (Step-by-Step), aimed at a slow relaxation of the confinement measures. It was devised as a five-stage program: Lockdown, Transition, Preparation, Early Reopening, Advanced Reopening, each with specific restrictions and obligations for individuals. The progress from one stage to the next is gradual, and each municipality will be centrally assigned to each stage according to their epidemiological statistics, with continuous monitoring of those indicators in order to allow them to progress or return between stages. On July 28th, the plan was finally implemented, where seven municipalities in RM and two others in the Valparaíso region exited Lockdown to be assigned to Transition. This policy slowed down the previous downward trend in the number of new cases.

With this reality in mind, we set ourselves to forecast and model the evolution of the disease in Chile. Several studies have modeled and predicted the spread of COVID-19 in different countries, using data that begins with the first reported cases. Some studies (6, 7) have fit data using Gaussian models or other standard regression models, which are inappropriate given the nature of discrete count data.

Phenomenological models (8–13) have been previously applied to various infectious disease outbreaks including other respiratory illnesses, such as severe acute respiratory syndrome (SARS) and pandemic influenza. These models, including the sub-epidemic growth model, can capture empirical patterns of past epidemics, and are useful in generating short-term forecasts of the daily trajectory of the epidemic. These approaches are especially useful when epidemiological data are limited. Real-time short-term forecasts generated from such models can be useful to guide the allocation of resources that are critical to bring the epidemic under control. Remuzzi and Remuzzi (14) used exponential growth models to predict the early propagation of the virus in Italy. Canals et al. (3) also used an exponential growth model to predict in the case of Chile. Maier and Brockmann (15) used sub-exponential growth in confirmed cases of recent COVID-19 outbreak in Mainland China. Exponential growth models, however, are unrealistic in scenarios where additional information is available: Once an epidemic has progressed, and mitigation measures start to have effects, contagion rates are slowed down, with a reduction of the count of new cases, making exponential growth models less appropriate for modeling purposes. Hence, logistic growth models are a better option to model data in these instances. For example, Roosa et al. (16), Aviv-Sharon and Aharoni (17), and Chen et al. (18) have used generalized logistic growth models and the Richards model (19) to generate forecasts of the cumulative reported cases of COVID-19 in China, Asia, and USA, respectively.

For our study, we have used a novel multi-parametric method that extends the standard logistic growth curve, allowing us to understand the past and predict the future evolution of the disease in Chile. The model is a nonlinear quasi-Poisson regression specification that explicitly accounts for overdispersion of the count data. The trend has been estimated using a Richards growth curve, incorporating weekday-specific effects and policy interventions as control variables. This sort of specification has not been used so far in previous COVID-19 studies. In specific, our approach allows for additional flexibility compared to other studies that analyze the evolution of COVID-19 in Chile [e.g, (3, 20)]. That additional flexibility of our specification allows us to both forecast and simulate multiple alternative scenarios, such as the continuation of the Lockdown policy (in contrast to the *Step-by-Step* policy) and changes in the growth rate of the epidemic.

In section 2, we describe our data, our forecasting methodology and model; in section 3, we present our estimation results; in section 4, we discuss our findings and conclude. In the Supplementary Material document, we offer an Appendix with additional comparative statics for different scenarios (with additional tables and results included as well).

## 2. Data and Methods

### 2.1. Description of the Data

Data used in this work comes from the epidemiological reports from the CMoH, spanning from February 28 until September 15, 2020. These epidemiological reports are updated overtime by the independent expert panel working with the CMoH, and it is updated regularly to adjust for errors and misreports. These most accurate counts are collected on the Chilean Ministry of Science (CMoSc) website at https://www.minciencia.gob.cl/covid19. Considering the many issues regarding collection and publication of COVID-19 data in Chile, we believe that this data source is the best option available to analyze the Chilean case, as other authors have similarly done [e.g., (3, 20, 21)].

The dataset includes the total count of confirmed cases according to (a) the date that COVID-19 symptoms first appeared (as provided by the patient), and (b) Polymerase chain reaction test (PCR) prognosis notification date (as registered by the physician on the CMoH surveillance system). It is important to mention that this case count is retroactively corrected as new cases are confirmed and the epidemiological situation evolves as measured by the CMoH epidemiological department. In this study, we decided to use the daily count of cases according to PCR notification date, due to higher reliability.

Since our study is based on secondary data from the CMoH's official daily public reports as published by the CMoSc, it did not require approval from an Ethics Committee.

### 2.2. Richards Growth Curve Models

The Richards growth curve model (19), a generalization of the logistic curve (22), is a growth curve model for population studies used in cases where growth is not symmetrical about the point of inflection (23, 24). It has been widely used to describe epidemiological processes for real-time prediction of outbreak of diseases [e.g., SARS (25), dengue (26), influenza H1N1 (27), and COVID-19 (7, 18)].

$$\begin{array}{l} \Lambda_{t}=\frac{K}{{\left(1+\exp\left(-r\left(t-t_{m}\right)\right)\right)}^{\alpha }}. \end{array}$$

Here, *K* is a parameter corresponding to the total count of infected people by the end of the pandemic, *r* is the daily hazard (infection) rate, *t*_*m*_ is the lag phase, and α is a variable which fixes the point of inflection and control asymmetry parameter. The first derivative of this function with respect to time *t* allows us to model the number of new cases.

$$\begin{array}{l} \lambda_{t}=\alpha rK\frac{\exp\left(-r\left(t-t_{m}\right)\right)}{{\left(1+\exp\left(-r\left(t-t_{m}\right)\right)\right)}^{\alpha +1}}. \end{array}$$

### 2.3. The Quasi-Poisson Approach

Expanding upon the logistic asymmetric Richards curve discussed previously, we have fitted a *Generalized Quasi-Poisson Nonlinear Regression* to model the evolution of daily cases, using explanatory covariates, to predict the daily number of COVID-19 cases in Chile. Poisson regressions are models used to model count data, assuming that the response variable is Poisson distributed. Denote {*Y*_*t*_} as the number of confirmed COVID-19 cases at time *t*, {**X**_*t*_} the vector of collected covariates at time *t*, and $F_{t-1}:=\left\{{\text{X}}_{t},Y_{t-1},{\text{X}}_{t-1},\ldots \right\}$ a collection of all realizations of {**X**} and {*Y*} until period *t*−1. The Poisson regression assumes that the response variable, conditional on the past, follows the following probability model:

$$\begin{array}{l} \mathrm{Pr}\left(Y_{t}=y|F_{t-1}\right)=\frac{\lambda_{t}^{y}\exp\left(-\lambda_{t}\right)}{y!},\;y=0,1,2,\ldots \end{array}$$

where $\text{E}\left(Y_{t}|F_{t-1}\right)=\text{Var}\left(Y_{t}|F_{t-1}\right)=\lambda_{t}>0$. Here, rate $\lambda_{t}=g\left(F_{t-1},\beta \right)$ is a function of the covariates, and of unknown β parameters to be estimated. If *g*(·) is a linear combination of the β parameters, then the model is considered a *Poisson Generalized Linear Model*.

A key assumption for the validity of Poisson regression models is that both the mean and the variance are the same. In our case, the variance is larger than the mean. We address this using a quasi-Poisson regression. In this model, count data is assumed as generated by an exponential family distribution where the variance is equal to the mean multiplied by an over-dispersion parameter ϕ > 1, thus,

$$\begin{array}{l} \text{ E}\left(Y_{t}|F_{t-1}\right)=\lambda_{t}\\ \text{Var}\left(Y_{t}|F_{t-1}\right)=\phi \lambda_{t} \end{array}$$

In our proposed model, covariates collected in {**X**_*t*_} include a weekday seasonal effect as well as holiday dummies. Both of these are crucial, considering that most PCR testing labs do not operate on weekends or holidays. Additionally, our proposal considers an intervention variable to capture the *Step-by-Step* confinement reduction policy, added to the Richards curve estimate.

(1) $$\begin{array}{l} \lambda_{t}=\left(\vartheta_{1}\frac{\exp\left(-\vartheta_{2}\left(t-\vartheta_{3}\right)\right)}{{\left(1+\exp\left(-\vartheta_{2}\left(t-\vartheta_{3}\right)\right)\right)}^{\vartheta_{4}}}+\exp\left(\psi \delta_{t}\right)\right)\\ \;\times \exp\left(\alpha_{1}{\text{Holiday}}_{t}+\overset{7}{\underset{j=1}{\sum }}\beta_{j}{\text{WeekDay}}_{j,t}\right), \end{array}$$

for *t* = 1, 2, …, 201, where WeekDay_*j,t*_ is a dummy variable equal to one when *t* corresponds to day *j*, *j* ∈ {Monday, ..., Sunday}; Holiday_*t*_ equals one if *t* is a holiday; δ_*t*_ is a dummy variable equal to one for *t* starting on July 28, 2020 (marking the start of the *Step-by-Step* policy); ϑ_1_ = α*rK*, ϑ_2_ = *r*, ϑ_3_ = *t*_*m*_ and ϑ_4_ = α + 1. Please note that, unlike the case of traditional linear regressions, in the quasi-Poisson model the estimated parameters do not have a direct *elasticity* or *marginal effect* interpretation. The parameters are estimated maximizing the quasi-likelihood function:

(2) $$\begin{array}{l} \hat{\theta }=\arg\underset{\theta }{\max}\overset{n}{\underset{t=1}{\sum }}\left[\frac{1}{\phi }y_{t}\log\left(\lambda_{t}\right)-\lambda_{t}+\kappa \left(y_{t},\phi \right)\right], \end{array}$$

where κ(*y*, ϕ) = ϕ^−1^[−*y* log(*y*) + *y*] and θ = (α_1_, β_1_, …, β_7_, ϑ_1_, …, ϑ_4_, ψ, ϕ).

As such, the model allows us to obtain an estimate for the basic reproduction number, *R*_0_(*t*).

(3) $$\begin{array}{l} R_{0}\left(t\right)=\frac{\hat{\lambda_{t}}}{\sum_{j=0}^{t-1}\hat{\lambda_{j}}} \end{array}$$

### 2.4. Implementation of Modeling Analysis

To estimate the parameters of the generalized linear model given by expression (1), we used the software R. The "gnm" library includes the function **gnm()**. The iterative algorithm requires starting values for the parameters, which were obtained through the function **nls()** in the "nls" library. To obtain confidence intervals for out-of-sample predictions, we approximated the quasi-Poisson likelihood with negative binomial distributions via a bootstrap of size 10,000, using the **ciTools** library in R, as described by (28). The intervals are, thus, built as follows:

1. The model in Equation (1) is fitted to obtain estimates $\hat{\theta }$ and $\hat{\text{Cov}}\left(\hat{\theta }\right)$. The number of simulations is set at 10,000.
2. Simulate 10,000 draws of the coefficients ${\hat{\theta }}_{*}\sim N\left(\hat{\theta },\hat{\text{Cov}}\left(\hat{\theta }\right)\right)$.
3. Simulate $Y_{*}|F_{t-1}$ from the response distribution using the following approximation:
  $$\text{Negative Binomial}\left(\hat{\lambda_{t}},\hat{\lambda_{t}}/\left(\hat{\phi }-1\right)\right).$$
4. Determine quantiles α/2 and (1 − α/2) of the simulated conditional responses.

Because information is updated daily, for replication purposes, full data was updated in our spreadsheet on September 16, 2020. For the actual realizations of data from that date until October 30, 2020, we used the November 7, 2020 update.

## 3. Results

The model was fitted to the observed daily cases from February 28 to September 15. Table 1 summarizes the estimated parameters, standard errors (SE), and 95% confidence intervals (CI). Parameters β_4_-β_5_ were statistically significant at *p* < 0.05, and all other parameters were significant at *p* < 0.001. The point estimate of the quasi-Poisson over-dispersion parameter is $\hat{\phi }=68.79$.

**Table 1:** Summary of estimated parameters.

**Parameter**	**Estimate**	**SE**	**Z**	***p*-value**	**Lower 95% CI**	**Upper 95% CI**
ϑ_1_	36695.16	5346	6.864	<0.0001	26216.723	47173.596
ϑ_2_	0.046	0.003	16.081	<0.0001	0.041	0.052
ϑ_3_	81.893	5.754	14.233	<0.0001	70.616	93.171
ϑ_4_	3.158	0.4102	7.698	<0.0001	2.354	3.962
ψ	7.166	0.081	87.772	<0.0001	7.006	7.326
α_1_	−0.564	0.089	−6.315	<0.0001	−0.739	−0.389
β_1_	0.268	0.030	8.951	<0.0001	0.209	0.326
β_2_	0.163	0.031	5.337	<0.0001	0.103	0.224
β_3_	0.113	0.032	3.571	<0.0001	0.051	0.176
β_4_	0.081	0.033	2.027	0.0158	0.016	0.147
β_5_	0.077	0.033	2.346	0.020	0.013	0.141
β_6_	−0.306	0.040	−7.733	<0.0001	−0.383	−0.228
β_7_	−0.507	0.043	−11.704	<0.0001	−0.591	−0.422

*Parameters estimated based on daily cases of COVID-19 in Chile between February 28, 2020 and September 15, 2020*.

**Figure 1:**
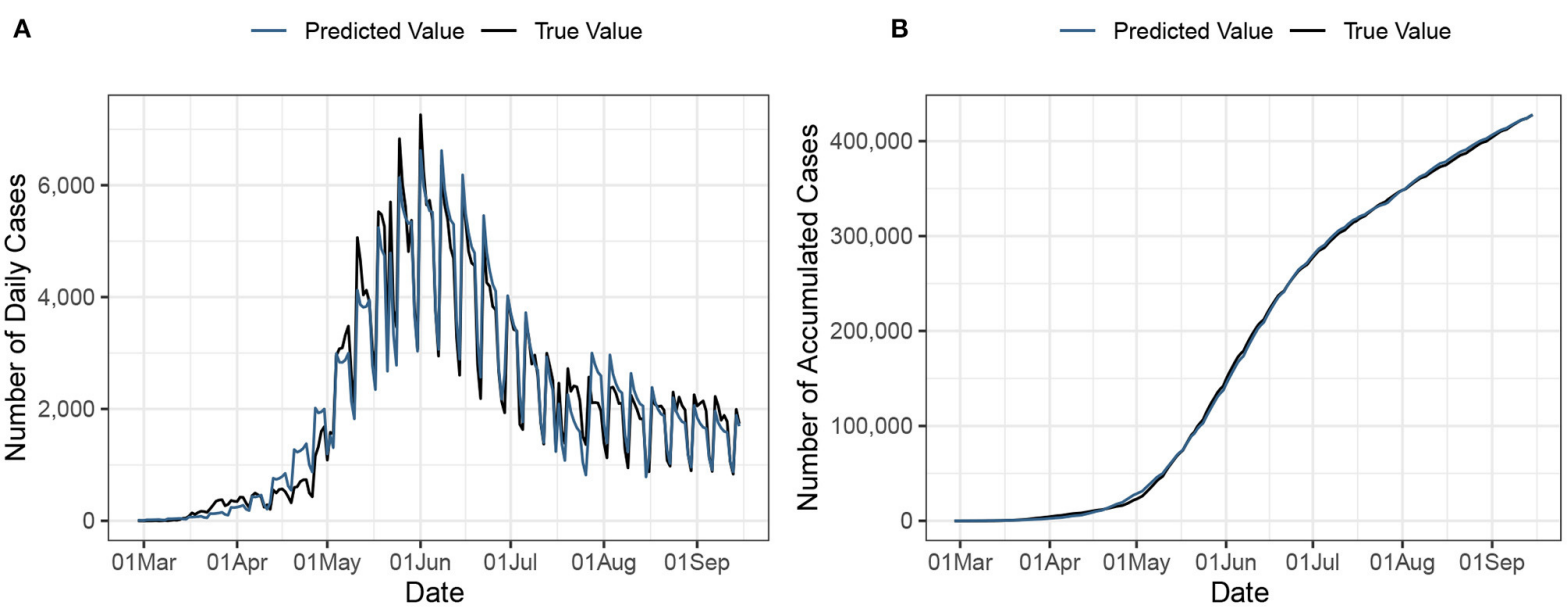
**(A)** Daily count of confirmed COVID-19 cases in Chile. **(B)** Cumulative count of COVID-19 cases in Chile.

For further illustration, Figure 1 offers a depiction of how well our estimated model fits the data. On Figure 1A, we display the daily number of new COVID-19 cases in Chile, with true values denoted with the black line, and fitted values denoted by the blue line. Analogously, Figure 1B showcases the cumulative count of COVID-19 cases in Chile, with the black line denoting the true cumulative count, and the blue line denoting fitted values. Accordingly, the goodness of fit of the model is assessed with the Heinzl-Mittlböck Pseudo *R*^2^ (29). The Pseudo *R*^2^ equals 95.3%, confirming the excellent fit of the model to the data.

Note that the proposed model offers a good visual fit to the evolution of the epidemic, where the curve succeeds at capturing the exponential growth of the data as well as the seasonal effects corresponding to weekdays/weekends.

The intervention variable captures the (statistically significant) slowdown of the decay effect typically present toward the end on Richards curves, as caused by the introduction of the *Step-by-Step* policy.

**Figure 2:**
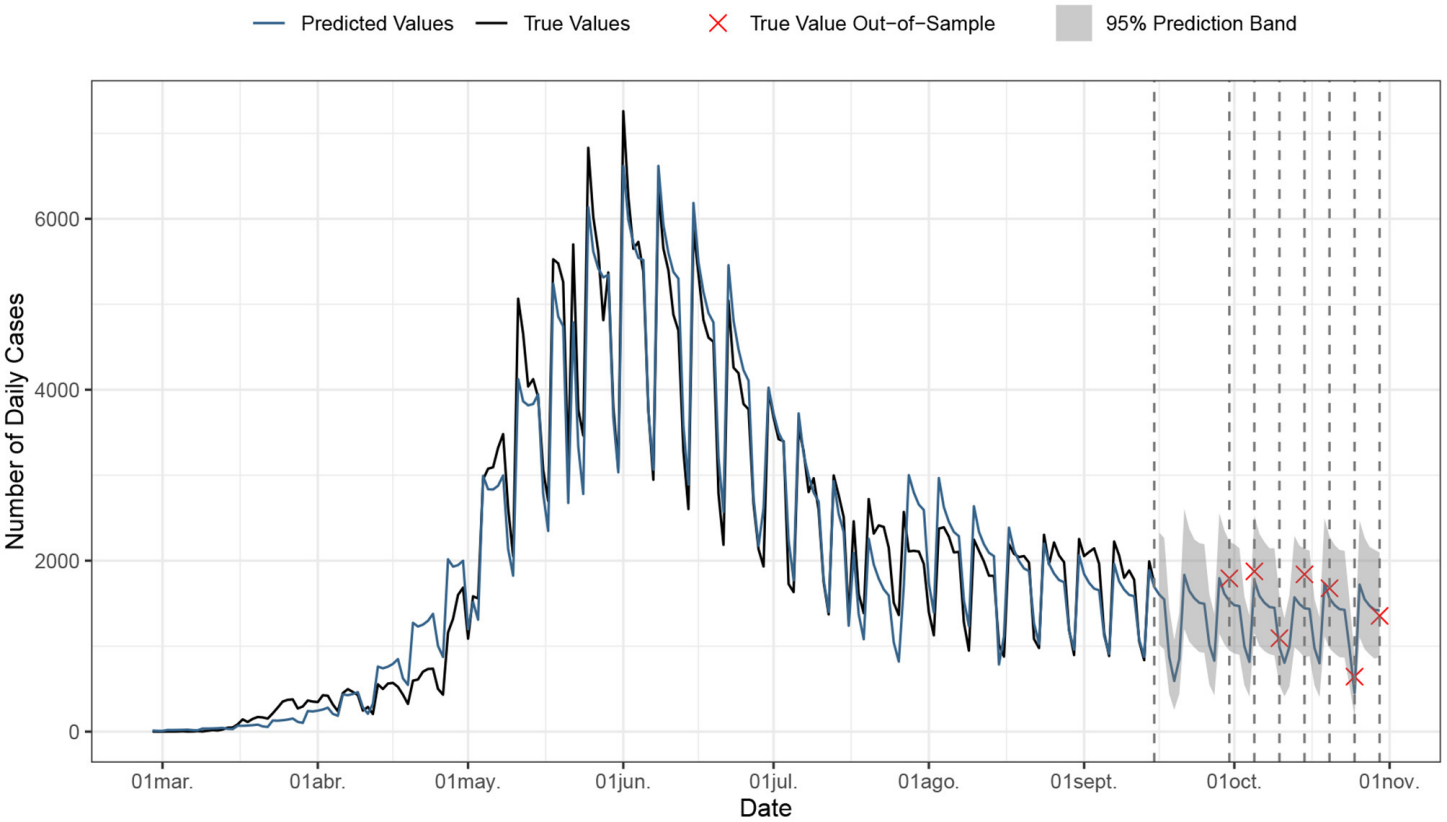
Forecast for future cases until October 30th.

Figure 2 shows our projected forecasts (together with 95% CI) for the short and medium run (up until October 30). Table 2 displays the values of the predicted new cases for specific dates of Figure 2, between September 16 and October 30, including 95% CI. Please note that, as can be observed from reading Table 2, all our predicted daily cases fall within the 95% CI, until the end of October 2020. Our predicted total number of cases at that date was almost 488,000 cases, against the actual count of 498,466 observed that day.

**Table 2:** Prediction of the fitted model.

**Date**	**Day of the week**	**Predicted cases**		**Daily cases CI**		**Actual cases**	
		**Daily**	**Accumulated**	**Lower 95%**	**Upper 95%**	**Daily**	**Accumulated**
2020-09-30	Wednesday	1530.5	448012.2	955.975	2218.025	1,793	454,948
2020-10-05	Monday	1766.4	454541.5	1134.000	2500.025	1,875	461,949
2020-10-10	Saturday	986.9	461532.4	548.000	1575.000	1,093	469,912
2020-10-15	Thursday	1443.4	467838.0	888.000	2136.000	1,840	477,559
2020-10-20	Tuesday	1558.1	474338.4	986.975	2249.025	1,677	484,464
2020-10-25	Sunday	451.4	480098.6	169.000	876.000	643	490,539
2020-10-30	Friday	1417.1	487681.7	868.000	2109.025	1,354	498,466

## 4. Discussion and Conclusions

The estimated model evidences that the trend of the data changes once the *Step-by-Step* governmental policy was implemented. Before that date, the daily count of new cases was decreasing at a faster rate than after the policy. This is a measure of the effects that the implementation or removal of lockdown policies have in the infection curve in the middle-to-short-run, understanding that new policy measures are likely to cause structural changes to the shape of the curve.

From the proposed model, we can observe that the estimated daily growth rate of COVID-19 in Chile is about 4.5% (95% CI: [4%, 5%]). Compared the to the rates observed in the US (16.9%, 95% CI: [15.9%, 17.8%]) (18) and China (17.12%) (30), we can conclude that the infection rate in Chile is about three times smaller. The growth rate in Chile in the second half of September 2020 implies that the cumulative count of COVID-19 cases in Chile doubles every 2 weeks.

The accuracy of our model, naturally, is contingent on the governmental level policy decisions as they emerge. The restrictive lockdown policies imposed by the CMoH in the RM starting on May 15 had an impact in the slow down of the epidemic's spread two weeks later (the disease's incubation period): We predict, using our model, that the count of COVID-19 cases would have been 491,096 by July 28 without lockdown policies, compared to the actual count (with lockdown in place) of 267,846 cases. This reduction in the total count made possible for the government to launch the *Step-by-Step* policy.

The *Step-by-Step* policy generates a break point in the downward trend observed before July 28. We forecast that the introduction of that change might increase the daily count of new cases up to ten times the expected count under lockdown. The short run impact is thus relevant, particularly considering that our model doesn't consider the possibility of a second outbreak of the disease, which in practice cannot be ruled out from happening. Indeed, a natural limitation of our study is the fact that the COVID-19 epidemic is still under development. Thus, the estimates of the parameters of the model have a substantial amount of uncertainty associated to them. For instance, after the end of the epidemic, the interpretation of the parameter *K* is the count of infected individuals at the end of the pandemic, which is not an entirely valid interpretation for our current data.

Our ability to understand the COVID-19 epidemic is essential in order to curb its global spread. Our study provides an important framework to inform public health decision-making designed to end the epidemic in different regions, by not only aiding decision makers in Chile, but also illustrating the usefulness of the quasi-Poisson modeling approach to follow the evolution of the disease when availability of data is limited.

Our selection of the growth model was based on intensive testing of other models: This functional specification produced the most accurate results, provided enough flexibility, and generated key information, including the exponential growth rate, the doubling time for the epidemic, and the effect of the governmental policy interventions in the level at which the rate of growth of the epidemic levels off. Our key contribution is methodological: We strongly believe that models from the generalized logistic family, such as the one we present here, are useful to be able to track the future trends of diseases like COVID-19.

Naturally, our study has limitations. Particularly, the time frame of the study corresponds to the data available until September 2020, and in the long run, it doesn't account for further interventions (as they indeed took place: Authorities implemented further relaxations of the containment measures in November and December, causing the growth rates of the epidemic to continue to increase until the date of this revision in March 2021).

One of the main limitations of the proposed approach is the fact that its ability to make accurate predictions only works for the short-term, being unclear in general for how long such predictions remain reasonably accurate. However, it is also fair to recognize that this limitation is not unique to the proposed approach, as any other model-based methods that serve similar purposes also present the same limitation. Table 2 shows in its final column the actual observed data, to make comparisons for the out-of-sample observations: In each case, the true observed daily cases fell within the 95% forecast confidence intervals (as we also displayed in Figure 2). As expected, the model's short-term daily forecast of new cases is close to the observed new cases. All in all, given the aforementioned reasons, long-term inferences using this type of model should not be considered. In spite of its limitations, findings from this study provide useful information to inform public health decision-making and policies designed to end the epidemic.

From a policy perspective, as Hodgins and Saad (31) noted, the high-income countries' blueprint of suppression and maintenance is less likely to be effective in low-and-middle-income countries. In specific, strict lockdowns like the ones implemented in Chile have had substantial negative impacts on the economy, access to education, and disruption of routine clinical services. This was the motivation behind the introduction of policies like *Step-by-Step*. It is unrealistic that radical suppression can be considered a viable policy in the long run. Tools like the one we present in our article, however, enable policy makers to keep a close eye on the evolution of the disease. In any case, it is crucial that authorities understand that the relaxation of protective measures caused by policy announcements such as the *Vacation Permits* released since December 2020 for the Summer Season in 2020/2021 had a direct effect on the count of new COVID-19 cases, as reflected by the upsurge of new cases present throughout the first trimester of 2021 in Chile.

## Data Availability Statement

The original dataset used in the study is included in the Supplementary Material (ZIP file), further inquiries can be directed to the corresponding author.

## Author Contributions

MV, CV, and BQ conducted all forecasts and data analyses, wrote the text of the manuscript in September 2020, and revised subsequent corrections to the document in March 2021. All authors read and approved the final manuscript.

## Conflict of Interest

The authors declare that the research was conducted in the absence of any commercial or financial relationships that could be construed as a potential conflict of interest.
