# Supplementary material for: Forecasting the 2020 COVID-19 Epidemic: A Multivariate Quasi-Poisson Regression to Model the Evolution of New Cases in Chile
Source: Front Public Health. 2021 Apr 23;9:610479. doi: 10.3389/fpubh.2021.610479 (PMC8102770; doi:10.3389/fpubh.2021.610479)
Supplement: Supplementary file 1 [file Data_Sheet_1.PDF]

Supplementary Materials for ”Forecasting the 2020 COVID-19 epidemic: A multivariate Quasi-Poisson regression to model the evolution of new cases in Chile”

Original article citation:
Vicuña, M.I; Vásquez, C.; Quiroga, B.F. (2021). ”Forecasting the 2020 COVID-19 epidemic: A multivariate Quasi-Poisson regression to model the evolution of new cases in Chile”. *Frontiers In Public Health*.

Comparative Statics: Intervention Analysis

Simulated Scenario A: *No Step-by-Step policy*

In Section 3, we described the evolution of new COVID-19 cases in Chile between February 28 and September 15, 2020. Between May 15 and July 27, most geographical areas (municipalities) of the country were subject to a full lockdown policy, with nightly curfews, and the issuance of travel permits for essential activities (as determined by the authorities) only.

This lockdown policy was replaced on July 28 with the *Step-by-Step* policy, to gradually move out of lockdown in select municipalities. In this section, we simulate what would have happened if the *Step-by-Step* policy had never been implemented, and the country had remained in full lockdown (*No Step-by-Step policy* scenario). Figure S1 illustrates the comparison of the two situations, with a forecast window defined between September 16 and October 30, 2020. In terms of parameters, the *No Step-by-Step* scenario is, thus, equivalent to restricting ψ to 0. The first segmented vertical line in Figure S1 corresponds to September 15, 2020, the end of the sample used in the estimations. Each of the subsequent segmented vertical lines denote the dates estimated in the simulations in the corresponding Table S1.

The baseline model forecasts that Chile would have accumulated about 448,000 infected cases by September 30 (the actual count was 450,250 by September 30), whereas for the model assuming that lockdown had never been lifted, the simulation predicted a count of 380,229 accumulated cases. This means that the *Step-by-Step* policy accounts for an increase of about 15%. By October 30, using our model fitted until mid September, that difference was predicted to be of about 21% (Predicted scenario = 370,799; Predicted baseline model = 487,682; Actual value = 498,466). Table S1 displays the simulated results under the *No Step-by-Step* policy scenario. Analyzing the % of accumulated efficiency, the model projected an increase of 10.5% in the total accumulative count of cases until October 30, 2020, compared to the count under the simulated *No Step-by-Step policy* scenario.

In simple terms, our simulation predicts that, if in Chile the *Step-by-Step* policy had not been implemented back in late July 2020, *ceteris paribus*, the country would have observed about 120,000 less COVID-19 positive cases by the end of October.

Table S1. Prediction of Number of Daily and Cumulative Cases in the *No Step-by-Step* Scenario

Date	Baseline		No Step-by-Step Scenario		Efficiency	Efficiency
	Daily	Accumulated	Daily	Accumulated	% Daily	% Accumulated
2020-09-30	1530.50	448012.2	93.99	369456.0	93.9	17.5
2020-10-05	1766.43	454541.5	88.25	369812.3	95.0	18.6
2020-10-10	986.88	461532.4	39.95	370121.6	96.0	19.8
2020-10-15	1443.42	467838.0	46.37	370341.2	96.8	20.8
2020-10-20	1558.05	474338.4	40.82	370525.8	97.4	21.9
2020-10-25	451.43	480098.6	9.28	370659.3	97.9	22.8
2020-10-30	1417.13	487681.7	23.63	370798.7	98.3	24.0

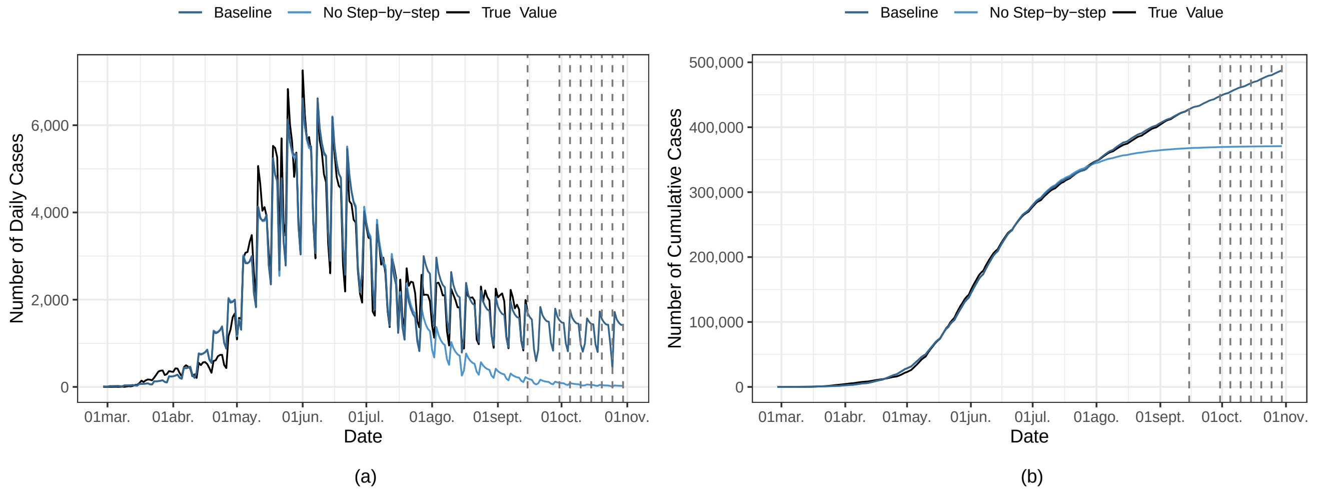

Figure S1. (a): Comparison of Number of the daily Cases in the *No Step-by-Step* Scenario. (b): Comparison of Cumulative Confirmed Cases in the *No Step-by-Step* Scenario. The first dotted line is the end of the estimation sample (Sept. 15), and the others are the dates on the corresponding Table S1.

Simulated Scenario B: Changes in the Epidemic Growth Rate

Our second scenario simulates what would happen with the growth rate of the Richards Curve after a certain point in time. Specifically, it is assumed that a threshold t_0 alters the parameters of the Richards Curve growth rate, Λ_t. Structurally r and λ are assumed as constant before and after the t_0 threshold. Parameter α controls the flatness of the curve of cumulative cases. Finally, $K^{'}$ is determined by the constraint: $K^{'} = \Lambda_t(t_0, K, \alpha, r, \lambda)(1 + e^{-r(t_0-\lambda)})^{\alpha^{'}}$. Thus:

$$\Lambda_t \quad = \quad \begin{cases} \Lambda_t(t, K, \alpha, r, \lambda) & \text{if } t \leq t_0 \\ \Lambda_t(t, K^{'}, \alpha^{'}, r, \lambda) & \text{if } t > t_0 \end{cases} \tag{S1}$$

where $\Lambda_t(t, K, \alpha, r, \lambda)$ is the Richards Curve defined in expression (1), assuming that $K^{'}/K > 1$, and that the parametric change preserves the continuity of Λ_t with respect to t at t_0:

$$\Lambda_t(t_0, K, \alpha, r, \lambda) = \Lambda_t(t_0, K^{'}, \alpha^{'}, r^{'}, \lambda^{'}) \tag{S2}$$

To better understand the intervention upon α, we define the *freedom intervention efficiency* as the relative increase on the total count of cases at the end of the epidemic, $\eta(t_0) = \frac{K'-K}{K} > 0$, by gradually increasing

freedom of circulation. In that sense, different levels of η are a consequence of different levels of α', each reflecting different possible de-confinement policies that an authority might undertake. By inducing different levels of α', we can compare those against the baseline where $\eta = 0\%$.

For this intervention, we assume that the mean of the Richards Curve changed after September 16 (hence, $t_0 = 202$). We evaluate three sub-scenarios: $\eta = 5\%$ (Scenario 1), $\eta = 10\%$ (Scenario 2), $\eta = 15\%$ (Scenario 3), with each value signaling an increased freedom of circulation. Figures S2 and S3 show the forecast for the daily count and cumulative count, respectively. Note how panel (a) in each case shows the full time range under consideration, whereas panel (b) shows the same results as (a) for a shorter time span to better observe the simulated predictions under each of the scenarios. Table S2 displays the forecasts under the baseline model and the three alternative scenarios. As before, the first segmented vertical line in Figure S2-S3 corresponds to September 15, 2020, the end of the sample used in the estimations. Each of the subsequent segmented vertical lines denote the dates estimated in the simulations in the corresponding Table S2.

In each of the three sub-scenarios, we observe that the higher the η parameter, the lower the efficiency of the freedom intervention. Naturally, this is an artificially-determined change in the rate, made for simulation purposes. In the real world, the parameter would not be a direct choice variable for the authorities, but an outcome estimate based on possible policy interventions. In the case of Chile, these increases could be the product of, for instance, the announcement of policies like *Holiday Celebration Plans* and *Vacation Permits*, as implemented beginning in December 2020. Those situations could help to explain, with a newly-fitted model, the upsurge of new cases beginning in December, in what has been colloquially labeled as "COVID-19's second wave."

Table S2. Intervention Analysis of the Daily and Cumulative Cases of Covid-19 in Chile.

Date	Baseline		Scenario 1		Scenario 2		Scenario 3	
	Daily	Accumulated	Daily	Accumulated	Daily	Accumulated	Daily	Accumulated
2020-09-30	1530.5	448012.2	1881.6	450970.5	2236.9	453983.8	2595.9	457047.9
2020-10-05	1766.4	454541.5	2206.2	459083.9	2650.0	463697.2	3097.4	468375.3
2020-10-10	986.9	461532.4	1194.6	467696.2	1405.5	473950.1	1619.3	480286.1
2020-10-15	1443.4	467838.0	1687.1	475152.2	1936.0	482579.0	2189.8	490108.6
2020-10-20	1558.1	474338.4	1768.5	482608.0	1984.5	491014.0	2205.8	499544.9
2020-10-25	451.4	480098.6	500.1	489062.6	550.3	498183.0	601.9	507446.9
2020-10-30	1417.1	487681.7	1539.0	497364.3	1665.0	507226.6	1794.8	517254.5

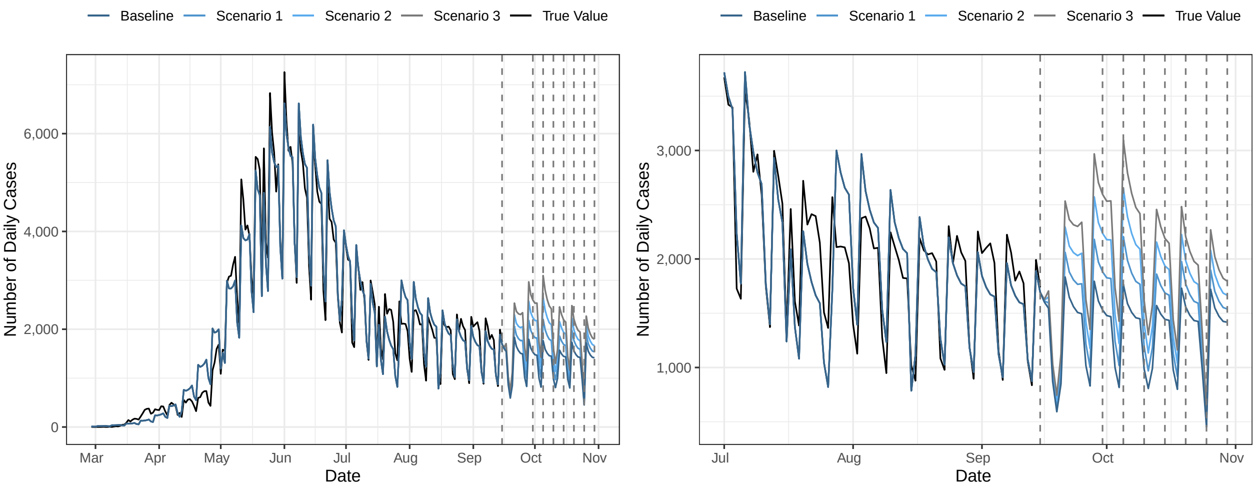

(a) (b)

Figure S2. (a): Intervention analysis of Daily Confirmed Cases of Covid-19 in Chile. (b): Intervention analysis of Daily Confirmed Cases of Covid-19 in Chile from July 1st to Oct. 30th 2020. The first dotted line is the end of the estimation sample (Sept. 15), and the others are the dates on the corresponding Table S2.

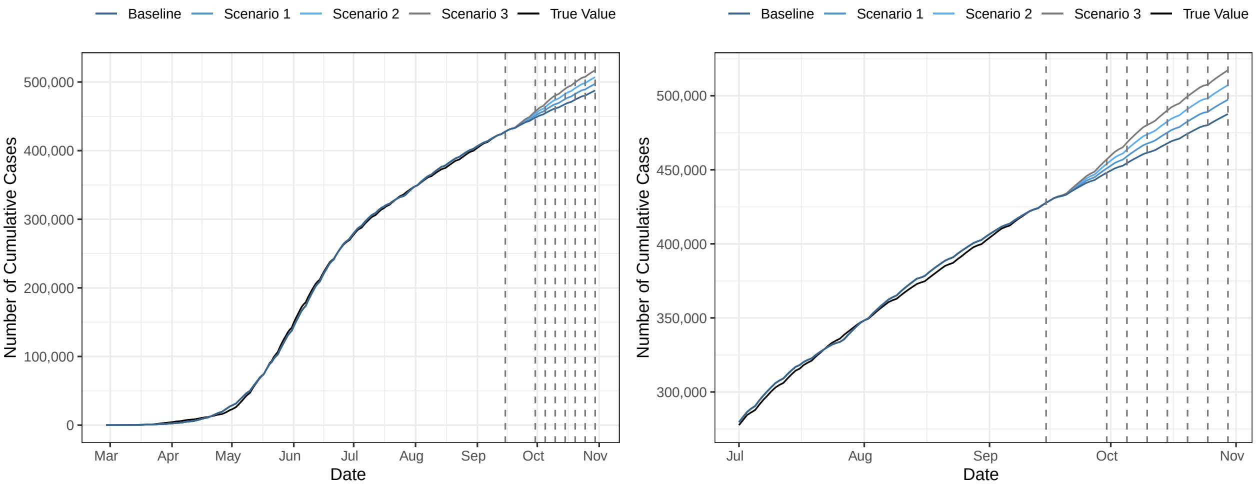

(a) (b)

Figure S3. (a): Intervention analysis of Cumulative Cases of Covid-19 in Chile. (b): Intervention analysis of of Cumulative Cases of Covid-19 in Chile from July 1st to Oct. 30th 2020. The first dotted line is the end of the estimation sample (Sept. 15), and the others are the dates on the corresponding Table S2.
